# Progress in State Medicine

**Published:** 1897-07-31

**Authors:** 


### PROGRESS IN STATE MEDICINE.

**Isolation Hospitals.**—Galsworthy[1] reviews the work of the London Metropolitan Asylums Board. There are eight hospitals open, accommodating 5,000 patients, two additional hospitals are in process of construction, and a site has been acquired for a third. The average cost of a hospital of 500 beds is £200,000. During the year 1896, 46,158 cases of infectious disease were notified in London, and of these the board have treated in their hospitals nearly 21,000, including 15,064 cases of scarlet-fever and 4,889 of diphtheria. The Board have also under their care the harmless insane poor. Its net expenditure last year amounted to £553,975.

Johnston[2] attributes the progressive annual reduction of the cases of zymotic disease in Glasgow, during the past 25 years, to the system of isolation pursued. With improved administration and nursing the mortality in the City Hospital from scarlet-fever has fallen from 17 per cent. to 4·9 per cent. Wards must be in separate, one-storied pavilions, and reserved for one type of disease. The shape should be oblong, capable of accommodating 20 to 30 beds. The best arrangement is two wards of 12 beds each, opening from a common corridor, length 72 ft., width 25 ft, height 14 ft. Seven windows on each side, at one end the bath-room, and at the other the lavatories in turrets. Floors of polished oak, wall of cement with glossy enamelled surfaces, fresh air inlets at floor level. A common kitchen is placed between the two wards. Heating by double-faced open fireplaces, and by low-pressure hot-water pipes. Electric lighting. Pavilions should be in rows, running north-east and south-west, and should not communicate by covered passages. Cooking by steam and gas is best conducted in a central kitchen, near the store-rooms, and provision must be made for laundries, stables, mortuary, and administrative quarters. The risk of treating scarlet fever, measles, and diphtheria on the same site is so great that he concludes that this practice should be abandoned. A receiving ward is necessary to prevent admission of doubtful or unsuitable cases; and these, together with cases of double infection, must be treated in special two-bedded wards. That a large number of patients are sent to hospitals certified as suffering from various diseases, but subsequently are found not to be so suffering, is shown by a recent report of the Metropolitan Asylums Board.[3] During 1895 and 1896 425 cases in which there was no typhoid were sent to their hospitals as suffering from that disease, and during the same period 1,200 cases wrongly diagnosed as diphtheria were admitted. It is satisfactory to find that only one patient developed typhoid, and 26 diphtheria subsequent to admission. The value and necessity of an early bacteriological examination is obvious from these facts. Brownlee and Dittmar[4] describe the method of procedure in the treatment of undefined cases as practised in the hospitals of the City of Glasgow. Each case is, as far as possible, isolated in a separate ward, or, if two or more cases are together, the cubic space per bed is never less than 4,000 cubic feet. They are here watched for four weeks, and at the end of that period, unless desquamation occur, are discharged. No dismissed case returned, or proved a means of further infection. No case contracted fever in the wards. Of 45 patients, 11 proved to be cases of scarlet fever, 6 were doubtful, and the other 28 were suffering from tonsilitis, measles, rötheln, dermatitis, cellulitis, and tuberculosis. M'Neill,[5] from the Local Government Board reports, shows that 9·1 per 1,000 of the rural population of Scotland suffer each year from one or other of the notifiable diseases, and that this figure approximates very closely to that of the towns, viz., 10·2 per 1,000. Whilst in the town 43 per cent. of the notified cases were isolated, in the country districts not more than 15 per cent., and sometimes only 3 per cent., of notified cases are isolated. The country districts are thus a source of danger to the towns, for communication between the two is incessant. The establishment of rural isolation hospitals would, therefore, not only benefit the particular neighbourhood, but also adjacent centres of population. With a properly constructed ambulance carriage, cases requiring isolation may be removed twelve to fifteen miles. The rural hospital should be placed in the most densely populated spot of the district. At least a provision of one bed per 1,000 of the resident population should be made, and the wards must be arranged so that it is possible to isolate two diseases simultaneously. Where it is possible, burghal and rural authorities should combine to support a joint institution. Temporary buildings of corrugated iron, huts, tents, &c., are condemned as expensive in the long run and inefficient. A sunny, dry, sheltered site, with reasonable facilities for drainage, should be chosen, and the number of patients must not exceed twenty per acre. Ward refuse and infectious excreta may be mixed with sawdust, saturated with paraffin, and burned; sewage, chemically treated in settling tanks, or purified by land filters. Recks' disinfecting apparatus may be employed where economy has to be considered. The administration should be in the hands of a special committee, and if possible all the cases should be attended by one medical man. A nursing staff might be kept constantly available by paying a retaining fee to a nurse's institute.

Graucher and Thoinot,[6] reporting for the Commission appointed by the Assistance Publique on hospital accommodation for tuberculous patients, estimate that one-third of the population is attacked by tuberculous disease, and that one-sixth succumb. In view of its undoubted contagiousness, and the great benefit derived from treatment, it is to the interest of both public and patient that the sufferers should be isolated. On no account should tuberculous patients be placed in a mixed ward, for not only may they prove a source of infection, but in such a situation it is impossible to provide ample ventilation day and night, or to secure the necessary amount of nourishment, rest, and sleep. Amongst the measures recommended are: (1) The substitution of washing for the present dry and dusty methods of cleaning; (2) the provision of spittoons in wards and corridors, which, with their contents, shall be systematically disinfected by boiling with soda solutions; (3) all articles used by patients—spoons, forks, glasses, plates, &c.—to be boiled in soda solutions; all linen, clothing, and bedding to undergo steam disinfection; (4) patients to be instructed in habits of greater personal cleanliness, frequent washings of hands and face; (5) mattresses to be of wood fibre; all furniture of simple construction to admit of easy disinfection; (6) the careful selection of healthy and suitable attendants.

**Milk.**—Hart[7] reports 95 outbreaks of disease traced to the agency of milk, 48 of typhoid, 32 of scarlatina, and 15 of diphtheria. These are in addition to the 73 outbreaks reported in 1881. A new feature in the present series is due to the recognition of the fact that disease of the cow herself may render the milk infective. In some outbreaks the occurrence of multiple diseases in the same place and even in the same individual was noticed. Whilst the filth conditions generally played a part in the causation of contamination, it was more common to discover cases of infectious disease that had been concealed, unnotified, or undetected, and in more than one case there were found amongst the dairy-workers persons who were in charge of sick patients. The amount of disease varied fairly constantly with the number of children per household, and with the quantity of unboiled milk consumed. Stringy or ropy condition of milk was noted in connection with several of the outbreaks, and the milk of cows recently calved seems especially liable to cause disease. This last is a very important discovery, outbursts of scarlatina, diphtheria, and enteric fever having been traced to this source. Klein's observations upon the eruptive diseases of milch cows and the spread of infection by their milk are fully borne out by many of the instances cited, both of scarlet fever and diphtheria. Epidemics are traced to the washing of cans with filthy water, and to the admixture of water through leaks in defective cooling apparatus. Separated milk and ice creams also play a part in the causation. Amongst remedial measures the chief is boiling the milk. The vendor or farmer should, however, be compelled to have at hand the means of scalding the cans, and an unpolluted supply of water is essential. Drainage and filth removal must receive more attention, and cleanliness in housing and milking the cattle be insisted upon. A simple and immediate method of stopping the supply is needed, and further powers of inspection should be granted to medical officers. The milk of cows suffering from tuberculosis, or from eruptive diseases, and of newly-calved cows should not be sold. Compulsory notification of the diseases of cattle, increased penalties for infringement of the present law, and some regulation of the ice cream trade are also needed. Freeman[8] tabulates 72 epidemics due to milk occurring between 1880-96, none of which are included in Hart's lists. Of these 53 were enteric fever, 11 diphtheria, 2 foot and mouth disease, 3 throat affections, and 3 intestinal disorders. He points out that milk is generally 36 hours old when used, and that during this time it has had ample opportunity for germ contamination, by far the greater number coming from the persons of the cow and milker.

[1] Rev. of Poor Law Administration in London, Times, Dec. 1896. [2] Infectious Diseases Hospitals, their Construction and Management, Jour. of State Med., Mar. 1897. [3] Rep. of General Purposes Committee, Feb., 1897. [4] Lancet, April 3, 1897. [5] Caled. Med. Journ., Jan. 1897. [6] Ann. d'Hygiene, xxxvi., 12. [7] Brit. Med. Journ., May 8, 1897. [8] Med. Rec., Mar. 28, 1897.

